# Microphysiological System‐Generated Physiological Shear Forces Reduce TNF‐α‐Mediated Cartilage Damage in a 3D Model of Arthritis

**DOI:** 10.1002/advs.202412010

**Published:** 2024-12-24

**Authors:** Alexandra Damerau, Duc Ha Do Nguyen, Christina Lubahn, Kasper Renggli, Moritz Pfeiffenberger, Gerhard Krönke, Matthias Herrmann, Thomas Leeuw, Frank Buttgereit, Timo Gaber

**Affiliations:** ^1^ Department of Rheumatology and Clinical Immunology Charité – Universitätsmedizin Berlin, Corporate Member of Freie Universität Berlin and Humboldt‐Universität zu Berlin 10117 Berlin Germany; ^2^ German Rheumatism Research Centre (DRFZ) Berlin a Leibniz Institute 10117 Berlin Germany; ^3^ School of Life Sciences University of Applied Sciences and Arts Northwestern Switzerland Muttenz 4132 Switzerland; ^4^ Immunology & Inflammation Research TA Sanofi‐Aventis Deutschland GmbH 65926 Frankfurt Germany

**Keywords:** articular cartilage breakdown, chondrogenic 3D model, immune cells, matrix degeneration, mesenchymal stromal cell, microfluidic system, pro‐inflammatory cytokine, TNF‐α

## Abstract

Osteoarthritis (OA) is a leading cause of disability, often resulting from overuse or injury, but inactivity can also contribute to cartilage degeneration. Conventional in vivo models struggle to isolate and study the specific effects of mechanical stress on cartilage health. To address this limitation, a microphysiological system (MPS) is established to examine how varying levels of shear stress impact cartilage homeostasis. The system allows for the cultivation of 3D chondrogenic microconstructs (CMCs) derived from human mesenchymal stromal cells, simulating both physiological and pathophysiological shear stress. Inflammation is induced via TNF‐α or activated peripheral blood mononuclear cells to model cartilage damage, enabling the evaluation of therapeutic interventions. The study demonstrates the development of an arthritis‐like phenotype and successful restoration of cartilage conditions through a JAK inhibitor under physiological shear stress. Physiological shear stress is identified as a critical factor in maintaining cartilage integrity. This MPS offers a standardized method to study shear stress, replicate cytokine‐induced cartilage damage, and simulate key features of arthritis, providing a valuable alternative to animal models.

## Graphical abstract

Osteoarthritis (OA) arises from mechanical stress or inactivity, impacting cartilage health. A microphysiological system (MPS) to investigate shear stress effects on 3D chondrogenic constructs, modeling cartilage damage with TNF‐α or immune cells is established. Physiological shear stress preserved construct integrity under these conditions, while therapeutic interventions restored cartilage health, offering a platform for preclinical drug testing.

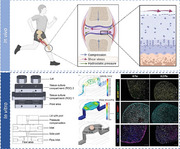

## 1 Introduction

Progressive cartilage degradation and subchondral bone destruction are hallmarks of two major joint diseases: osteoarthritis (OA) and rheumatoid arthritis (RA). Whereas OA is the most common degenerative joint disease (prevalence: 5%), RA represents the most common autoimmune‐mediated joint disease (prevalence: 0.5%–1%).^[^ ^1^ ^]^ The pathogenesis of RA is primarily characterized by pronounced systemic inflammation. In OA, osteophyte formation and synovial fibrosis are the most prominent.^[^ ^2^ ^]^ During the pathogenesis of both diseases, articular cartilage, which is naturally incapable of self‐regeneration, becomes damaged, degenerated, and ultimately degraded if not treated adequately.^[^ ^3^ ^]^ The increasing life expectancy and aging demographics exacerbate the enormous individual and economic burden imposed by these joint diseases.^[^ ^3a,b^ ^]^ Various factors are known to contribute to cartilage wear and tear, including aging, obesity, joint instability, excessive joint locomotion, trauma, and inflammation. Conversely, lack of physical activity and the non‐use of joints due to inability and confinement to bed results in low or so‐called hypophysiological shear stress and can also lead to irreparable cartilage damage. However, that knowledge has not yet translated into therapeutic approaches to restore damaged cartilage.^[^ ^4^ ^]^

Although the aforementioned factors are linked to compressive and shear stress, these stresses at physiological levels are also required to nourish the articular cartilage and maintain the physiology of cartilage, subchondral bone, and synovium.^[^ ^5^ ^]^ Articular cartilage is an extracellular matrix (ECM)‐rich tissue composed of 60%–80% water, 20% proteoglycans/aggrecans, 5% collagen (Col) type II, and 1%–5% chondrocytes. The movement of fluid within the cartilage is required to transport solutes. Therefore, physiological mechanical forces are essential.^[^ ^6^ ^]^ At the same time, compressive‐ and shear stress are the predominant mechanical stresses that lead to cartilage‐ and joint dysfunction, damage, and pain if they are too excessive.^[^ ^7^ ^]^ Physiological levels of compression and fluid shear stress positively impact articular cartilage by promoting chondrocyte proliferation, ECM production, growth, and differentiation.^[^ ^8^ ^]^ However, this mechanical stress can also have negative effects, such as chondrocyte death and the secretion of pro‐inflammatory factors when there is excessive compressive strain.^[^ ^9^ ^]^ Additionally, hypophysiological stress can lead to chondrocyte apoptosis and matrix degradation due to an insufficient supply of necessary mechanical stress.^[^ ^8^ ^]^

Animal models are invaluable for studying diseases of the entire joint and are rightly still the gold standard. It should be noted, however, that undeniable differences in cartilage thickness, anatomy, and biomechanical joint loading in animals compared to humans can lead to misleading results when investigating the influence of mechanical forces on cartilage damage.^[^ ^10^ ^]^ In particular, animal models are rather unsuitable for studying various mechanical forces and their effects on cartilage homeostasis in a defined and standardizable way, including compression and shear forces during pathological overuse and disuse. In preclinical studies, the application of the 3R principles and thus the development of physiological and pathophysiological in vitro systems is highly desirable.^[^ ^11^ ^]^ Therefore, microphysiological systems (MPSs) mimicking mechanical stress during joint locomotion on in vitro 3D human chondrogenic microconstructs (CMCs) have become increasingly important in preclinical research.^[^ ^12^ ^]^ These 3D CMCs integrate cells, soluble factors, and ECM‐like matrices, providing cell‐cell interactions and insights into matrix production and degradation to model articular cartilage. Of note, most 3D models involve scaffolds such as hyaluronic acid (HA),^[^ ^13^ ^]^ agarose,^[^ ^14^ ^]^ alginate,^[^ ^15^ ^]^ and hybrids such as HA/collagen,^[^ ^16^ ^]^ that affect seeded cells to a certain degree,^[^ ^17^ ^]^ scaffold‐free 3D pellet approaches are now used for both in vivo cartilage repair and in vitro modeling.^[^ ^18^ ^]^ Finally, to maintain the 3D architecture of CMCs and apply shear stress in a standardized manner, an MPS provides the opportunity to precisely control the environment through continuous perfusion, supply of soluble factors, and monitoring of pH, pO_2_, glucose, and lactate.

In this study, we focused on simulating shear stress specifically, as it is particularly relevant to pathophysiological conditions such as those observed in OA. Elevated shear stress often indicates abnormal loading scenarios, such as those arising when joint surfaces do not glide optimally after an injury or in cases of overweight. Additionally, shear stress is the first type of mechanical load to be significantly reduced in bedridden patients, making it critical for understanding cartilage health under both normal and altered conditions. Therefore, we developed an MPS with an incubation chamber unit optimized to apply shear stress that results in a biological response that is similar to that observed in vivo during joint movement. In addition, our MPS supports the standardized in vitro development of human CMCs. We demonstrate that physiological shear stress protects the CMC against TNF‐α‐mediated arthritis, similar to the in vivo situation. On the other hand, pathologically low mechanical stress shifts the balance of matrix deposition and resorption toward catabolism upon TNF treatment; an effect that can be restored by JAK inhibition. These findings demonstrate that the combination of MPS and animal‐free in vitro model can be used as an effective preclinical tool to determine chondro‐metabolic cartilage alterations considering both physiological and pathological shear stress.

## 2 Results

### 2.1 Microphysiological System for Physiological Perfused Cultivation of 3D Chondrogenic Microconstructs

**Figure 1:**
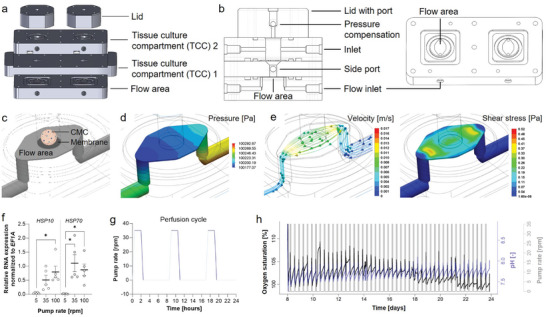
Design of the MPS to apply defined fluidic shear stress on CMCs. a) The MPS consists of dual‐compartment chambers based on polysulfone, featuring configurable geometries and separated by a polycarbonate membrane. b) Both compartments have channels for medium in‐ and out‐flow (inlets) and an integrated port‐lid‐system to withdraw medium or apply substances. Cross section and side view (left) and lower compartment in top view (right). c) Visualization of the CMC placed in the TCC1 cylinder on top of the membrane above the flow area. d) The pressure in Pa is shown with a pump rate of 35 rpm and shows a pressure difference of ≈100 Pa between the entrance of the flow chamber and the end. e) During the perfusion phase, a defined mechanical shear stress of ≈0.205 Pa is applied to the surface of the lower culture compartment as simulated with SolidWorks with a pump rate of 35 rpm. Fluid velocity is shown by lines with arrows with a medium velocity of ≈0.008 m ^−1^s. f) CMCs were cultivated with pump rates of 5, 35, and 100 rpm for a perfusion phase of 1.5 h. Relative gene expression of heat shock protein 10 (*HSP10*) and *HSP70* was normalized to the housekeeping gene elongation factor 1‐alpha 1 (*EF1A*). Statistical analysis was performed using the Friedman Test with Dunn's multiple comparison test g) Schematic overview of the intermittent perfusion cycle for 1.5 h three times daily representing the human locomotion and rest phase (6.5 h between cycles). h) Process monitoring of pump speed, pH, and oxygen saturation exemplary for one bioreactor run. pH is regulated by gassing with air and CO_2_ in regulated amounts. Process data were provided from the cloud web user interface of the OSPIN GmbH.

To set up an MPS for physiological perfused cultivation, we first developed a dual‐chamber system (**Figure** **1**a–c), which is integrated into a modular bioreactor platform (OSPIN GmbH, Berlin, Germany; Figure S2, Supporting Information). The novel dual‐chamber system consists of two dual tissue culture compartments (TCC) that allow applying standardized uniform defined mechanical shear stress (see Figure 1a,b). The dual‐chamber system consists of 5 components which were printed using polysulfone and assembled as shown in Figure 1a. Functionally, the TCC1 is spatially separated from the flow area below by a flexible polycarbonate membrane (8 µm pores with a density of 1000 pores per cm^2^, Neuro Probe Inc., Gaithersburg, USA) and from the TCC2 above it by a second membrane (Figure 1b). TCC1 was designed for fluidic shear stress experiments, while TCC2 focused on cytokine, immune cell, and drug interactions. Although TCC2 offers the opportunity to apply an additional flow rate, e.g., mimicking blood flow, we did not apply an additional media flow in our experimental setup.

Two ports, one in the lid and one on the side, allow the addition of cells/substances or the removal of samples in/from each TCC. The TCCs can be supplied via separate fluid paths to create media gradients and thus represent different microenvironments in vitro in each TCC. The medium flow below the TCC1 at a pumping rate of 35 rpm at 37 °C exerts a well‐defined shear stress on the cells/3D constructs in TCC1 (Figure 1b). In general, four chambers, consisting of two double chambers, can be operated simultaneously. Medium reservoirs were refilled daily to grant a constant medium supply.

The Kedem‐Katchalsky (K‐K) model predicts the volume flow (J_v_) and solute flow (J_s_) across the membrane as a function of the pressure difference (∆p) and, the osmotic pressure difference (∆Π) across the membrane. The (K‐K) equations for J_v_ and J_s_ are J_v_ = L_p_Δp – L_p_σΔΠ and J_s_ = ωΔΠ + (1−σ)c_mean_J_v_.^[^ ^19^ ^]^ Where (σ) is the reflection coefficient, (L_p_) is the hydraulic permeability, and (ω) is the solute permeability. Assuming relatively uniform concentration and therefore moderate osmotic pressure, the K‐K model shows a linear correlation between the pressure difference and the volumetric flow rate J_v_ through the membrane. Basic 3D flow simulations of the flow area have shown a pressure drop between the entrance and the exit of the chamber (Figure 1c). The average pressure above TCC1 is fixed to the atmospheric pressure. At the same time, due to the membrane permeability, the average pressure within the flow area can be assumed to be approximately equal to the pressure in TCC1. This results approximately in a positive 100 Pa pressure difference between the flow area and TCC1 at the entrance of the flow chamber and a similar negative one at the end (Figure 1c). These pressure differences cause a media flow entering the tissue chamber and exiting it toward the end of the chamber. Note that the media circulation will reduce the predicted pressure drop in the chamber which in turn will modify the circulation itself. This, combined with other assumptions made and the complexities of the system, suggest that extra nonlinearities may occur in the J_v_ versus Δp relation for the present case (Figure S3, Supporting Information). With a membrane permeability of 1000 ml/(min∗cm^2^) at 10 psi, we can calculate the flow around CMC that sits onto of the membrane as J_v_ = L_p_Δp = 0.18 ml min^−1^.

Therefore, the mechanical stress acting on TCC1 was calculated according to the formula Re = (ρ∗vm∗l)/µ and 𝜏 = (6∗𝑄∗𝜇)/𝑤ℎ^2^ based on the values depicted in Table S3. The calculated shear stress in the flow chamber at a pumping rate of 35 rpm at 37 °C that the 3D construct directly sees above the membrane is 𝜏 = 0.15 Pa. This corresponds to ≈75% of the simulated (0.19 to 0.22 Pa) and calculated (0.2 Pa) shear stress below the membrane as obtained by the computer‐aided design (CAD) software SolidWorks 2021 (Dassault Systèmes, Waltham, USA). The assumptions for these considerations are a fluid temperature of 37 °C, laminar flow, an incompressible Newtonian fluid, atmospheric pressure, gravitational stress of 9.81 m ^−1^s^2^, and non‐slip wall boundaries (Figure 1d). The inlet was defined as a velocity inlet with a flow rate of 1.21∗10^−8^ m^3^/s and the outlet was set to atmospheric pressure. The velocity of the medium in the area of the TCC1 is ≈0.008 m^−1^s^−1^.

We first tested if the designed device is suitable for applying physiological and hypo‐ or hyperphysiological shear forces in the TCC1 that are capable of inducing a biological response of human MSC‐derived pre‐mature CMCs. Therefore, we applied shear forces at either 0 rpm (0 Pa = hypophysiological), 35 rpm (0.2 Pa = physiological), or 100 rpm (0.7 Pa = hyperphysiological) by intermittent perfusion cycle which ramps up for 1.5 h three times daily, with a resting period without perfusion of 6.5 h between each. In particular, we analyzed the transcriptional alterations of affected pre‐mature CMCs focusing on heat shock protein 10 (*HSP10*) and *HSP70* (Figure 1e) and other read‐outs that demonstrated a pump rate‐ and therefore shear force‐dependent response (Figure S4, Supporting Information). In addition, the system enables continuous monitoring and controlling of the pH value and tracking the oxygen partial pressure (pO_2_) during the perfusion cycles (Figure 1f,g). User control, process creation, monitoring, and process documentation were performed using a browser‐based user interface (OSPIN GmbH, Berlin, Germany). In our setup, the TCC1 served as an incubation chamber for the CMCs subjected to defined mechanical shear stress. Cytokine‐ and immune cell‐mediated cartilage damage mimicking synovitis was induced by adding either TNF‐α or stimulated PBMCs in the TCC2.

### 2.2 Physiological Shear Stress Results in Mature CMCs and Protects Against Chondrocyte Apoptosis and Matrix Degradation

**Figure 2:**
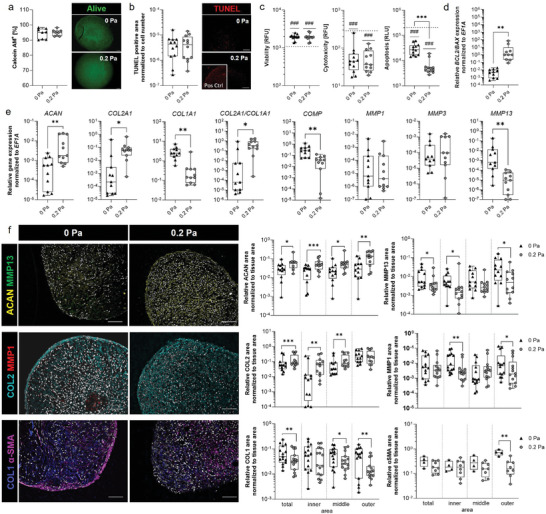
14 days of physiologic fluidic shear stress protects CMCs against cell apoptosis and matrix degradation resulting in mature 3D CMCs. a) Calcein‐AM staining was quantified using ImageJ (n = 8). b) TUNEL staining was performed to quantify apoptotic cells normalized to the cell number (DAPI staining) using ImageJ (n = 12). c) ApoTox‐Glo™ assay analyzing cell viability, cytotoxicity, and apoptosis level for n = 12. Dotted line for viability: 100 µg ml^−1^ Digitonin; cytotoxicity: 4% Triton X‐100; apoptosis: 0.1 mM Camptothecin. d) Relative expression of *BCL2/BAX* and e) cartilage‐related anabolic and catabolic marker genes normalized to the housekeeper *EF1A* (n = 11). f) Exemplary images for ACAN (yellow), MMP13 (green), COL2 (cyan), MMP1 (red), COL1 (blue), α‐SMA (magenta), and DAPI (gray) are shown. Scale bars represent 100 µm. Image quantification was performed to determine the stained area normalized to the tissue area (duplicates per data point) using ImageJ (n = 12; n = 4‐8 for αSMA). Data are shown as box plots (center line, median; box limits, upper and lower quartiles; whiskers, maximum and minimum values; all data points). Statistical analysis was performed using the Wilcoxon matched‐pairs signed rank test a–f), Mann‐Whitney U Test (f; αSMA) with *p < 0.05, **p < 0.01, ***p < 0.001, and Wilcoxon Signed Rank Test (c) with ^###^ *p* < 0.001.

After the successful establishment of fully animal‐free CMC culture conditions (Figure S5, Supporting Information) and demonstrating shear stress can be applied within the device, we initially used the MPS to establish long‐term stable mature CMCs by controlling the physiological environment and integrating perfusion. CMCs were pre‐maturated for 7 days in ULA‐plates. Subsequently, the CMCs underwent 14 days of mechanical stimulation resembling locomotion. Therefore, we exposed CMCs to fluidic shear stress in the MPS using hypophysiological (0 Pa) versus physiological (0.2 Pa) forces by applying repetitive cycles of 1.5 h perfusion and 6.5 h resting. Analyzing viability and apoptosis using LIVE/DEAD staining, TUNEL, and ApoTox‐Glo assay, we observed no influence on viability and cell death but a significantly reduced apoptosis as reflected by a decreased caspase activity and a higher *BCL2‐BAX*‐ratio (**Figure** **2**a–d). Next, we analyzed the gene expression of the ECM markers *ACAN*, *COL2A1*, *COL1A1*, *COMP*, and the matrix‐degrading enzymes *MMP1, MMP3, and MMP13*. We observed higher expression of *ACAN*, *COL2A1*, and a higher *COL2A1*‐to‐*COL1A1‐ratio* but lower expression of *COL1A1*, *COMP*, and *MMP13* at a fluidic shear force of 0.2 Pa – corresponding to physiological force conditions – compared to 0 Pa (Figure 2e). This indicated enhanced chondrogenesis, the formation of a loosened, non‐fibrotic ECM, and reduced ECM degradation (Figure 2e). On the protein level, CMCs display higher cartilage‐specific ECM deposits such as collagen types II (COL2) and aggrecan (ACAN), while collagen type I (COL1) and matrix‐degrading enzymes (MMP1 and MMP13) were less expressed at fluidic shear stress of 0.2 Pa compared to 0 Pa (Figure 2f). Of note, the profibrotic contractile actin isoform αsmooth muscle actin (αSMA) was less expressed at 0.2 Pa (Figure 2f). Interestingly, we observed distinct responses to mechanical stress across the inner, middle, and outer sections of the CMCs. Specifically, under physiological shear stress, anabolic chondrogenic ECM components, such as COL2 and ACAN, were predominantly expressed higher in the outer sections compared. Conversely, COL1 was expressed higher in the inner sections compared to the outer sections. Under physiological shear stress, catabolic MMP1 and MMP13 remained constant while being expressed higher at a fluidic shear stress of 0 Pa. Of note, profibrotic αSMA was only expressed higher at 0 Pa.

Our results suggest that incubation of CMCs under long‐term physiological shear stress conditions triggers the expression of anabolic genes, promotes chondrogenesis, and protects against chondrocyte apoptosis and matrix degradation differentially affecting the inner, middle, and outer area of the CMCs.

### 2.3 Physiological Shear Forces Reduce Apoptosis and IL‐6 Secretion in a TNF‐α Challenged CMC

**Figure 3:**
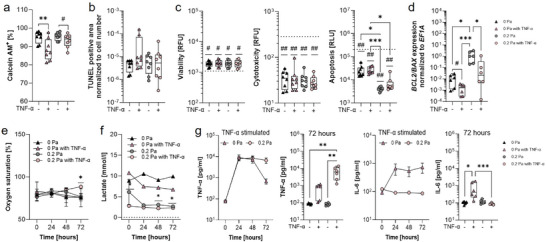
Short‐term exposure of 100 ng ml^−1^ TNF‐α for three days induced programmed cell death in CMCs. a) Calcein‐AM staining was performed after 21 days and quantified using ImageJ (n = 8). b) TUNEL staining was performed to quantify apoptotic cells normalized to the cell number (DAPI staining) using ImageJ (n = 8). c) ApoTox‐Glo™ assay analyzing cell viability, cytotoxicity, and apoptosis level for n = 8. Dotted line for viability: 100 µg ml^−1^ Digitonin; cytotoxicity: 4% Triton X‐100; apoptosis: 0.1 mM Camptothecin. d) Gene expression of *BCL2* and *BAX* normalized to the housekeeping gene *EF1A* (n = 6). e) Daily measurement of oxygen consumption using a Clark electrode (n = 6). f) Lactate concentration [mmol/l] within the supernatant was measured using the Biosen C‐line analyzer (n = 6). The control (dotted line: mean lactate = 0.24 mmol l^−1^) shows lactate concentration within the cell‐free culture medium. g) Quantification of the cytokine levels of TNF‐α and soluble IL‐6, comparing 0 Pa and 0.2 Pa shear stress and the unstimulated and TNFα stimulated CMCs (n = 6). Data a‐d are shown in box plots (centerline, median; box limits, upper and lower quartiles; whiskers, maximum and minimum values) and e‐h are shown in symbols with mean and SEM. Statistical analysis was performed using the Friedman test with Dunn's multiple comparisons test (a–d; g) and the Mixed‐effects model with the Geisser‐Greenhouse correction and Tukey's multiple comparisons test (e–*g*). *P*‐values are indicated in the graphs with ^#^ *p* < 0.1, *p < 0.05, **p < 0.01, ***p < 0.001. Wilcoxon Signed Rank Test was performed to the control (c). *P*‐values are indicated in the graph with ^#^ *p* < 0.05, ^##^ *p* < 0.01.

A fundamental clinical experience is that moderate targeted and regular exercise therapy is vital for most rheumatic diseases including OA and RA as a lack of physical movement can lead to irreparable cartilage damage. Therefore, we hypothesized that physiological shear force conditions mimicking joint locomotion should protect our established mature CMCs against TNF‐α‐mediated damage if our in vitro model can reflect arthritic changes and is, therefore, suitable to mimic mechanical influences within the joint. For this reason, after 21 days of maturation, we stimulated CMCs with TNF‐α for 6 h every day in the afternoon for three days during the resting phase, which corresponds to the circadian cytokine peaks in the body. After each TNF‐α treatment, CMCs were left untreated for 18 h. TNF‐α was administered at a concentration of 100 ng ml^−1^, which corresponds to a non‐cytotoxic but intensive proinflammatory stimulation to achieve maximum effects within a short observational period of three days. TNF‐α stimulation significantly reduced the relative number of Calcein‐AM^+^ cells as a measure of cell survival but to a lower extent at 0.2 Pa mimicking joint movement (**Figure** **3**a). However, using TUNEL staining, viability, cytotoxicity, and apoptosis assays (Figure 3b,c), we did not observe any difference upon TNF‐α stimulation despite a numerical reduced density of cell nuclei per area at both 0 and 0.2 Pa (Figure S6a, Supporting Information). In addition, TNF‐α treatment at 0.2 Pa resulted in a significant decrease in the *BCL2‐BAX* ratio compared to the untreated 0.2 Pa group, which was still higher than the 0 Pa groups (Figure 3d). WST‐1 level increased upon TNF‐α treatment to a higher extent at 0 Pa than at 0.2 Pa demonstrating higher metabolic activity (Figure S6b, Supporting Information), while TNF‐α treatment at 0.2 Pa reduced oxygen uptake at 72 h compared to all other conditions analyzed (Figure 3e) and decreased lactate production by CMCs suggesting lower glucose uptake (Figure 3f).

Next, we analyzed the TNF‐α‐mediated IL‐6 secretion as a measure of the CMC's inflammatory response.^[^ ^20^ ^]^ TNF‐α was applied once a day for 6 h (= resting phase) and measured to monitor the TNF‐α uptake/release and IL‐6 secretion. The decrease in TNFα under 0 Pa conditions was accompanied by an increase in IL‐6 secretion over time, while significantly lower levels of the soluble pro‐inflammatory cytokine IL‐6 were observed under 0.2 Pa conditions (Figure 3g).

### 2.4 Physiological Shear Forces Maintain Matrix Homeostasis under TNA‐α Challenging and Protect Against Cartilage Degradation

**Figure 4:**
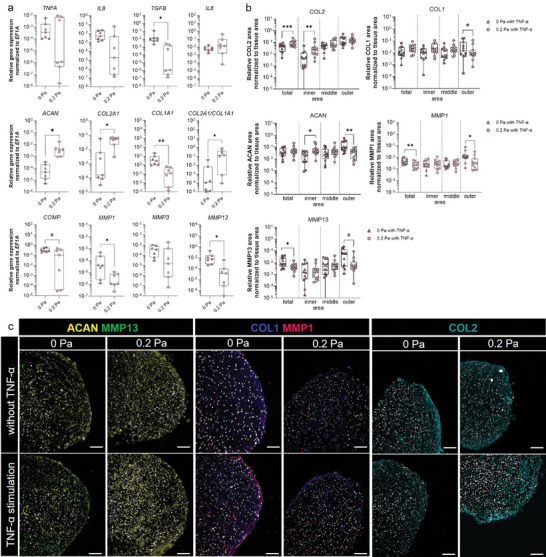
Physiological shear stress protects against TNF‐mediated CMC damage maintaining CMC homeostasis while hypophysiological shear stress supports TNF‐mediated CMC damage. a) Gene expression of inflammatory, anabolic, and catabolic marker genes was performed after total RNA extraction using SYBR Green and normalized to the housekeeping gene *EF1A* (n = 7). b) Image quantification was performed to determine the stained area normalized to the tissue area (duplicates per data point) using ImageJ (n = 11). c) Exemplary images for ACAN (yellow), MMP13 (green), COL1 (blue), MMP1 (magenta), COL2 (cyan), and DAPI (gray). Scale bars represent 100 µm. Data are shown as box plots (center line, median; box limits, upper and lower quartiles; whiskers, maximum and minimum values; all data points). Statistical analysis was performed using Wilcoxon matched‐pairs signed rank test with #*p* < 0.1, *p < 0.05, **p < 0.01, ***p < 0.001.

It is well‐known that TNF‐α causes degradation of both aggrecan and type II collagen by inducing the expression of MMPs.^[^ ^21^ ^]^ Therefore, we next investigated the influence of 0 and 0.2 Pa shear stress on TNF‐α‐mediated gene expression of the inflammatory *IL6*, *TNFA*, and *IL8*, the profibrotic *TGFB* and molecules involved in ECM turnover *ACAN*, *COL2A1*, *COL1A1*, *COMP*, *MMP1*, *MMP3*, and *MMP13* (**Figure** **4**a). Although *IL6*, *TNFA*, and *IL8* were not affected by the different shear stress, physiologic shear stress of 0.2 Pa reduced the profibrotic *TGFB* and *COMP* compared to hypophysiological forces. Of note, physiological conditions of 0.2 Pa showed a significantly higher expression of *ACAN*, *COL2A1*, and reduced *COL1A1* expression, thereby increasing the *COL2A1*‐to‐*COL1A1‐*ratio. Expression of *MMP1* and *MMP13* were significantly higher at 0 Pa after TNF‐α stimulation (Figure 4a). Using immunofluorescence analysis to identify the regions responsible for maintaining CMC physiology at 0.2 Pa, we observed that the inner region of CMCs is sensitive to changes induced by shear stress for the anabolic molecules of the ECM, COL2, and ACAN. The outer region is sensitive to the catabolic molecules of the ECM, namely MMP1 and MMP13 (Figure 4b,c). It is noteworthy that ACAN also appears to be affected in the outer region. These results indicate that the physiological shear stress of 0.2 Pa may maybe protective in terms of maintaining CMC homeostasis in our MPS, while hypophysiological shear stress of 0 Pa supports CMC damage.

### 2.5 Pharmacological Validation of the Arthritic CMC: Activated PBMCs Reduce the Expression of ACAN and COL2A1 and Promote MMP1 and MMP13, Which is Reversed by the JAK Inhibitor Baricitinib

**Figure 5:**
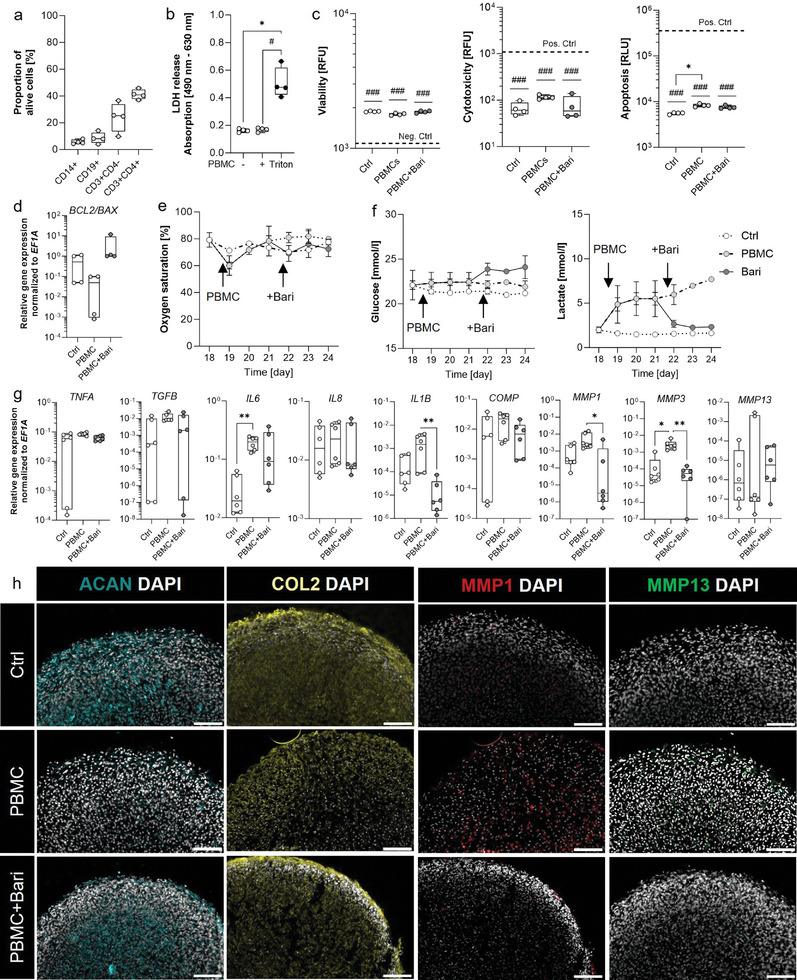
Activated PBMCs induce humoral‐mediated CMC damage, inflammatory mediators, and MMPs, which were attenuated by subsequent JAK inhibition. a) Proportion of alive PBMCs and composition of PBMCs (n = 4). b) Stimulation of the CMCs by activated PBMCs. LDH cytotoxicity assay was conducted from the supernatant after 24 h to analyze the cytotoxicity. High ctrl = 4% Triton^TM^ X100 for 24 h (n = 4). c) ApoTox‐Glo™ assay analyzing cell viability, cytotoxicity, and apoptosis level for n = 4. Dotted line for viability: 100 µg ml^−1^ Digitonin; cytotoxicity: 4% Triton X‐100; apoptosis: 0.1 mM Camptothecin. d) Relative expression of *BCL2‐BAX* ratio and normalized to the housekeeper *EF1A* (n = 4). e) Daily measurement of oxygen consumption using a Clark electrode (n = 4‐8). f) Glucose and lactate concentration [mmol/l] within the supernatant was measured using the Biosen C‐line analyzer (n = 4–8). g) Relative expression of cartilage‐related anabolic and catabolic marker genes normalized to the housekeeper *EF1A* (n = 6). h) Exemplary images for ACAN (cyan), COL2 (yellow), MMP1 (red), MMP13 (green), and DAPI (gray) are shown (n = 4). Scale bars represent 100 µm. Data (a–d, g) are shown as box plots (center line, median; box limits, upper and lower quartiles; whiskers, maximum and minimum values; all data points) and e‐f are shown in symbols with mean and SEM. Statistical analysis was performed using the Friedman test (b,c), Kruskal‐Wallis test with Dunn's multiple comparisons test (d,g), and Mixed‐effects analysis applying the Geisser‐Greenhouse correction along with Tukey's multiple comparison test performed to the control (e,f). *P*‐values are indicated in the graphs with ^#^ *p* < 0.1, *p < 0.05, **p < 0.01. Wilcoxon Signed Rank Test was performed to the control (c). *P*‐values are indicated in the graph with ^###^ *p* < 0.001.

Initially, we achieved mature CMCs after a maturation period of 7 days followed by exposure to physiological shear stress for 14 days in three parallel experimental setups. In two of these experiments, we subsequently added activated PBMCs to the upper compartment of the culture chamber to simulate humoral immune cell‐mediated cartilage damage of mature CMCs under a physiological shear stress of 0.2 Pa for 6 days. This approach allowed us to closely mimic the conditions of humoral immune cell‐mediated cartilage damage in our experimental setup. To standardize our approach, we only used PBMCs from donors with a similar frequency to the main cell populations (**Figure** **5**a). In one of them, we treated the PBMCs and CMCs after three days for additional three days with the JAK inhibitor (JAKi) baricitinib, a small molecule used to treat patients with moderate to severe RA by inhibiting JAK1 and JAK2 as approved by the Food and Drug Administration (FDA).^[^ ^22^ ^]^

First, we confirmed that the addition of activated PBMCs or their combination with baricitinib did not affect the viability, cytotoxicity, or apoptosis of the CMCs (Figure 5b,c). Secondly, we revealed a PBMC‐mediated significant induction of caspase 3/7 activity (Figure 5c) and a decrease in *BCL2*‐to‐*BAX*‐ratio (Figure 5d), which was reversed by baricitinib treatment. In the initial phase of cell‐mediated joint inflammation, immune cells only promote apoptosis in the outer areas of cartilage as these cells are in close contact with the synovial fluid and synovial membrane resembling our results (Figure S7, Supporting Information). The addition of activated PBMCs led to an altered metabolic rate of chondrocytes and immune cells. While the oxygen saturation of the medium initially decreased, it increased after two days compared to untreated CMCs. The addition of baricitinib restored the oxygen saturation of the medium (Figure 5e). Of note, a decrease in oxygen saturation of the medium indicates an increase in cellular oxygen consumption. The amount of glucose in the medium decreased less in CMCs treated with activated PBMCs than in CMCs alone, but more than in CMCs treated with activated PBMCs and the addition of baricitinib (Figure 5f). The amount of lactate increased over time when CMCs were treated with activated PBMCs. Conversely, the addition of baricitinib resulted in lactate levels similar to that observed in CMCs without treatment (Figure 5f).

On the gene expression level, immune cell‐mediated activation of CMCs significantly increased the expression of *IL6* and *IL1B* as well as matrix‐degrading enzymes that lead to the key phenomenon of RA ─ cartilage degradation (Figure 5g). These effects could be also attenuated by JAK inhibition. The results observed on the mRNA level were also reflected on the protein level as analyzed by immunofluorescence staining. Morphologically, the outer area containing elongated cells disappeared with stimulation using activated PBMCs, thereby displaying signs of maceration of the superficial cell layer (Figure 5h). In addition, an increased expression of MMP1 and MMP13 leading to the breakdown of cartilage ECM was induced by immune cells and abolished by baricitinib (Figure 5h).

## 3 Discussion

The intricate architecture and specific functions of cartilage are of fundamental importance for the smooth functioning of joints in the human body. However, this avascular tissue is prone to inflammatory degenerative diseases, which include OA as one of the leading causes of disabilities worldwide. The complexity of OA, characterized by cartilage degradation, inflammation, and pain makes it challenging to study and treat. Conventional in vivo models, while invaluable, often fail to adequately replicate the multifactorial nature and dynamic progression of this disease. Mechanical stress such as compressive and shear stress can contribute to cartilage wear and tear if they are either too excessive or too low.^[^ ^8^, ^23^ ^]^ Shear stress in particular has been reported to be a critical component in the destructive profile of cartilage wear and tear.^[^ ^24^ ^]^ To mimic the shear stress observed in vivo during joint movements, we used an MPS and designed, engineered, and optimized a cultivation chamber unit. Using this approach, we i) developed a long‐term model of mature healthy CMCs and ii) accurately simulated shear stress in a physiological and pathophysiological manner. Furthermore, this approach enabled us to iii) mimic humoral and cellular cartilage damage to iv) test the efficacy of new therapeutics.

To this end, we have developed a microfluidic system that can keep cell constructs alive and sustain their chondrogenic phenotype under physiological conditions for at least three weeks. The modular bioreactor device of the company OSPIN GmbH served as the basic system for our MPS. We designed and fabricated a cultivation chamber to implement human‐derived CMCs. To carry out various investigations, we added ports for sampling and administration of stimulants at different times during the experiments. The MPS was further optimized to mechanically stimulate the cultured CMCs. The fluidic shear stress exerted on the cultivated CMCs was controlled via the pump rate mimicking shear stress during locomotion. In our calculation, the shear stress in the flow chamber was 0.20 Pa, which corresponds to the computer‐simulated shear stress of ≈0.19 to 0.22 Pa, of which 0.15 Pa acts directly on the CMC at a pumping rate of 35 rpm and 37 °C (Figure 1). The ability of mesenchymal cells to respond to mechanical stress is a hallmark for health and differentiation in the context of, e.g., tissue fibrosis and cartilage homeostasis.^[^ ^25^ ^]^ We demonstrated that our setup enabled shear rate‐dependent induction of *HSP10* and *HSP70* expression (Figure 1). This effect is consistent with the study by Xu and colleagues, who found that mechanical stress induces rapid activation of heat shock transcription factors.^[^ ^19^ ^]^ In addition, an accumulation of *HPS70* is induced, peaking between 6 and 12 h after cyclic shear stress exposure.^[^ ^19^ ^]^ Salibe‐Filho et al. exposed human lung endothelium to pathological (1.5 Pa) or physiological (0.5 Pa) shear stress, resulting in the expression of *HSP70* even under physiological shear stress.^[^ ^26^ ^]^ *HSP70* is sensitive to mechanical stressors, cytoprotective, and assists protein folding, transport, and assembly,^[^ ^27^ ^]^ while *HSP10* is a chaperonin for *HSP60* and, therefore, essential for its function.^[^ ^28^ ^]^ Thus, the experimental validation and the in silico simulation indicated excellent agreement with the mathematical calculation of the fluidic shear stress (Figure 1).

Next, we established standardized and reproducible conditions to generate animal‐free primary CMCs based on human bone marrow‐derived MSCs. The critical step in engineering functional chondrogenic cell constructs is the mesenchymal condensation initiating cell‐cell communication before chondrogenic differentiation.^[^ ^29^ ^]^ To achieve optimal cell‐cell contact, a sufficiently high cell density is essential for the initiation of chondrogenesis in vitro.^[^ ^29^, ^30^ ^]^ Tassey and colleagues demonstrated long‐term stability of the structure, viability, and phenotype of chondrogenic spheroids developed by using low‐attachment plates and medium supplemented with methylcellulose.^[^ ^31^ ^]^ Thus, to support the initial cell‐cell contact we used low‐attachment plates that resulted in the formation of viable 3D CMCs (Figure 2). To achieve long‐term viability, we repeatedly changed the medium supply and added perfusion‐mediated shear stress (Figure 2, 3, 4). As a result, we observed that perfused cultivation reduced apoptosis within the CMCs and supported chondrogenic differentiation. Cultivation at physiological 0.2 Pa yielded CMCs rich in aggrecan and collagen type II with decreased collagen type I expression (constitutive elements of the ECM of articular cartilage) confirming the observations in MSC‐based CMCs reported earlier.^[^ ^32^ ^]^ In addition, the perfused CMCs showed expression levels of maturation after 21 days that were comparable to our previously published mature macromolecular cartilage model.^[^ ^33^ ^]^ Consistent with other reports, our findings showed a decrease in the expression of the matrix‐degrading enzymes *MMP1* and *MMP13*.^[^ ^34^ ^]^ Interestingly, we observed distinct responses to mechanical stress across the inner, middle, and outer sections of the CMCs (Figure 2). Under physiological shear stress, the anabolic ECM component, collagen type II, was predominantly maintained in the different sections while being reduced at pathophysiological shear stress. Conversely, ECM‐stiffening collagen type I showed an opposite trend. However, this central zone was less affected by the catabolic activity of MMPs, suggesting a protective effect that supports cartilage integrity. The outer regions of the CMCs showed a higher expression of matrix‐degrading enzymes, particularly MMP1 and MMP13, at hypophysiological shear stress but lower at physiological shear stress. This effect aligns with the areas directly exposed to mechanical loading and potentially more vulnerable to external stressors. The spatial differences indicate that physiological shear stress helps maintain cartilage homeostasis by balancing matrix synthesis and degradation within the constructs. These findings underscore the importance of regional responses in evaluating cartilage health and suggest that stress modulation in the outer zones could play a key role in managing early degenerative changes in cartilage. Moreover, we and others demonstrated that during in vitro chondrogenesis of MSCs the expression of collagen type I and α‐SMA indicates the formation of fibrocartilage.^[^ ^35^ ^]^ Using our setup, we were able to prevent the formation of fibrocartilage by physiological mechanical stimulation, reducing the expression of collagen type I and α‐SMA. In the absence of perfusion, we observed cells with an elongated morphology in the outer zone, whereas under perfusion most cells had a more rounded shape, reflecting the optimal chondrocyte phenotype of cartilage.^[^ ^36^ ^]^ Thus, we conclude from these results that our in vitro model can reflect the importance of physiological mechanical stress to maintain cartilage homeostasis.

In the next step, we investigated whether physiological mechanical stress also alleviates early cytokine‐mediated catabolic features of cartilage degradation as seen in OA and RA. Mechanical abnormalities, e.g., due to overload from obesity or insufficient load from bed rest, increase catabolic effects and thus favor cartilage degradation.^[^ ^37^ ^]^ Using our in vitro approach, we administered TNF‐α – one of the most important pro‐inflammatory cytokines in arthritis^[^ ^38^ ^]^ – in a high but non‐cytotoxic dose (100 ng ml^−1^) during the respective non‐perfusing incubation phase for three days. Apart from driving inflammation, TNF‐α plays a pivotal role in cell apoptosis and tissue damage.^[^ ^39^ ^]^ Indeed, we observed a decrease in cell number and an increase in DNA fragmentation and caspase‐3/‐7‐activity upon TNF‐α treatment (Figure 5). Notably, caspase‐3‐mediated cell apoptosis and cartilage destruction is a well‐documented feature of OA and correlates with the disease severity.^[^ ^39^, ^40^ ^]^ In addition, we also show that the extent of TNF‐α‐mediated damage is less pronounced under conditions of physiological shear stress.

Furthermore, we observed minor metabolic changes during TNF‐α‐treatment but larger ones when comparing conditions with shear stress. While TNF‐α‐treatment resulted in higher enzymatic NADH turnover (measured by WST‐1), 0.2 Pa application reduced this turnover, reduced O_2_ uptake, and decreased glycolysis (assessed by the decrease in lactate concentrations). Finally, the metabolic calming of CMCs resulted in a reduction of differentially expressed proteins including the secretion of IL‐6 (Figure 5). We assume that this effect might be due to the previously reported inhibition of TNF‐α‐induced proinflammatory responses by physiological shear stress, e.g., in endothelial cells.^[^ ^41^ ^]^ Although we did not observe an inhibition of TNF‐α‐induced pro‐inflammatory gene expression, physiological shear stress inhibited profibrotic *TGFB*, preventing the formation of fibrocartilage.^[^ ^35^ ^]^ Again, physiological shear stress maintains cartilage homeostasis even in the presence of TNF‐α, as we have observed at the gene and protein level. While gene expression levels of matrix proteins including *ACAN* and *COL2A1* increased, matrix‐degrading *MMP1* and *MMP13* decreased in their expression. Previous studies have shown that MMP1, 3, and 13 are capable of degrading proteoglycans, while MMP1 and MMP13 specifically break down collagen types 1–4.^[^ ^42^ ^]^ TNF‐α‐induced MMP1 and MMP13 were predominantly expressed in the outer superficial layer, but to a much lesser extent under physiological shear stress. Moreover, TNF‐α‐mediated degradation of collagen type 2 in the outer superficial layer was more pronounced at 0 Pa than at 0.2 Pa, an effect reported previously by Wu et al.^[^ ^43^ ^]^ Our observations are consistent with a report on the expression of MMPs in the cartilage of rabbits after transection of the anterior cruciate ligament, a model for traumatic OA.^[^ ^44^ ^]^ The authors also observed the highest expression of MMP1 in the superficial zone of the cartilage.^[^ ^44^ ^]^ Conversely, MMP13 is predominantly expressed in the center of the CMCs, which is similar to the situation in vivo, where MMP13 is preferentially localized in the deep layer of human arthritic cartilage.^[^ ^45^ ^]^ We concluded from the similarities between our in vitro approach and the in vivo situation, that our approach is suitable to mimic TNF‐α‐ and shear stress‐mediated effects on cartilage physiology. Furthermore, we mimicked immune cell‐mediated cartilage damage using activated PBMCs at physiological shear stress in the presence and absence of therapeutic JAK inhibition. Using baricitinib an FDA‐ and European Medicines Agency (EMA)‐approved JAKi, we simulated the clinical situation of cartilage homeostasis in arthritis patients with and without therapeutic intervention. As expected, treating CMCs under physiological shear stress with activated PBMCs resulted in typical features of cartilage damage including the increase in CMC apoptosis, reduction in BCL2/BAX ratio, metabolic alterations (aerobic glycolysis and increase in oxygen consumption), increase in the expression of proinflammatory *IL6*, *IL1B*, and matrix‐degrading enzymes *MMP1*, and *MMP3*. Subsequent JAK inhibition with baricitinib impressively restored the physiological state of CMCs. *BCL2/BAX* ratio, aerobic glycolysis *IL1B*, and matrix‐degrading enzymes *MMP1*, and *MMP3* were restored reflecting therapeutic efficacy in our human in vitro model applying physiological shear stress that mimics joint movement. Interestingly, *MMP3* is significantly more expressed in the treated CMCs. MMP3 is known to activate other MMPs, such as proMMP1, and indicates its contribution to OA by activating latent collagenases.^[^ ^46^ ^]^ Furthermore, the cartilage‐destroying effect of activated immune cells and the clinical therapeutic efficacy of JAK inhibition are impressively demonstrated in the histological analyses of ACAN, COL2, MMP1, and MMP13.

Although we could demonstrate with our model the induction of disease phenotype and its restoration with therapeutic intervention, some experimental requirements and limitations remain that need to be addressed and optimized in the future. The most important experimental requirements and limitations include (i) the use of a high TNF‐α dose to achieve results within a short‐term exposure of three days, (ii) an artificial microenvironment to create optimal culture conditions that are usually hostile and characterized by hypoxia, and limited nutrient supply, (iii) the importance of maintaining consistent flow conditions for accurate calculations and interpretations and finally (iv) the relatively small size of our CMCs which does not restrict nutrient and oxygen supply but needs to be standardized throughout the experiments. In brief, we must acknowledge that the size and type of biological sample and the loading protocol, including rest periods and frequency, can significantly influence outcomes. Furthermore, adjustments to the loading protocol could yield different biological responses, potentially affecting chondroprotective effects or other targeted outcomes.

We conclude from our data that our experimental approach with a small‐sized scaffold‐free in vitro 3D chondrogenic model sourcing from human bone marrow‐derived MSCs can be used as an in vitro model to study cytokine‐driven cell‐ and matrix‐related changes during cartilage degradation, replicating a key feature of rheumatic joint diseases such as OA and RA. Furthermore, we established a bioreactor platform with adjustable tissue culture chambers that enables the study of the effects of mechanical stress and provides an intriguing platform to explore disease mechanisms or drug responses using organoids or other biological samples such as ex vivo cartilage explants from humans, human bodies, or immature and mature animals, provided the tissue complies with the model's parameters. With its adaptability, our MPS could support research on disease mechanisms, drug testing, and regenerative approaches in cartilage, providing a high degree of relevance to human cartilage biology with and without animal use.

## 4 Experimental Section

### Development and Perfused Cultivation of Scaffold‐Free Chondrogenic Microconstructs

Scaffold‐free CMCs were generated based on human bone marrow‐derived mesenchymal stromal cells (MSCs). MSCs were isolated from patients undergoing total hip replacement operations as previously described^[^ ^33^ ^]^ and provided by the Center for Musculoskeletal Surgery, Charité–Universitätsmedizin Berlin, and distributed by the “Tissue Harvesting” core facility of the Berlin‐Brandenburg Center for Regenerative Therapies. Study design and protocols were approved by the Charité‐Universitätsmedizin Ethics Committee and performed according to the Helsinki Declaration (EA1/012/13, January 2013, EA1/146/21, May 2021) and informed written consent was obtained from all human participants involved in the study. Subsequently, CMCs were generated by seeding 2∗10^5^ MSCs in 96‐well U‐bottom low‐attachment plates (Corning Inc., New York, USA), followed by a centrifugation step (400 *x g*, 4 min, RT). CMCs were left untreated within the first two days for self‐organized mesenchymal condensation. Afterward, the differentiation process was initiated by adding 100% StemMACS ChondroDiff (Miltenyi Biotec, Bergisch Gladbach, Germany) for one week. The CMCs were transferred into the tissue culture compartment 1 (TCC1) of the dual‐chamber system (Figure 1a–c) which was integrated into a modular bioreactor platform (OSPIN GmbH, Berlin, Germany; Figure S2, Supporting Information). CMCs were cultivated by intermittent perfusion cycle which ramps up for 1.5 h three times daily, with a resting period without perfusion of 6.5 h between each. Medium reservoirs with Dulbecco`s Modified Eagle Minimal Essential Medium (DMEM) GlutaMAX (Gibco, Waltham, USA) supplemented with 2% human platelet lysate (hPL) (PL BioScience GmbH, Aachen, Germany), 100 U ml^−1^ penicillin (Gibco, Waltham, USA), 100 µg ml^−1^ streptomycin (Gibco, Waltham, MA, USA), and 20% StemMACS ChondroDiff (named “experimental medium” from here on) were refilled daily to grant constant medium supply. Calibration of the InPro3253i pH probes and the InPro6850i pO_2_ probes was performed before each process using the Intelligent Sensor Management Software from Mettler‐Toledo GmbH (Gießen, Germany). For the pO_2_ probe, an external two‐point calibration with O_2_‐enriched cultivation medium and 2% sodium sulfite solution was also performed using the OSPIN software (OSPIN GmbH, Berlin, Germany).

### Immune Cell Isolation and Characterization

Peripheral blood mononuclear cells (PBMCs) were isolated by Ficoll‐Paque PLUS (GE Healthcare, Chicago, USA) density gradient centrifugation. Cell preparations were performed according to the manufacturer's instructions. Briefly, 20 ml blood was diluted with 17.5 ml phosphate‐buffered saline (PBS; Th. Geyer GmbH & Co. KG, Renningen, Germany)/bovine serum albumin (BSA; Sigma–Aldrich, Munich, Germany) at room temperature and carefully layered onto 12.5 ml Ficoll‐Paque Plus (Cytiva Sweden AB, Uppsala, Sweden), followed by a centrifugation step at 778 *x g* for 20 min at 20 °C without brakes. The layer of PBMCs was carefully aspirated and washed with 30 ml of cold PBS/BSA.

For the characterization, unspecific binding sites of the Fc‐receptors were first blocked using 10 mg ml^−1^ Flebogamma (IgG1 66.6%, IgG2 28.5%, IgG3 2.7%, IgG4 2.2%; Grifols, Barcelona, Spain) for 10 min on ice. Subsequently, cells were stained using antibodies from the German Rheumatism Research Center against CD3‐FITC (UCHT1, 1:200) and Miltenyi Biotec against CD4‐PE (REA623, 1:50), CD14‐APC (REA599, 1:50), CD19‐PerCP (LT19, 1:10), CD69‐PeVio770 (REA824, 1:50) for 20 min on ice. Cells were washed, centrifuged (400 *× g*, 4 min, 4 °C), and suspended in PBS/ 0.2% BSA/ 0.02% sodium azide (sodium azide from Sigma–Aldrich, Munich, Germany). Measurements were performed in a BD FACS CantoII device with FACSDiva software (BD Biosciences, Heidelberg, Germany) and analyzed with FlowJo software (version 10.7.1, Tree Star, Oregon, USA).

### Experimental Setup of Inflammatory Conditions

For TNF‐α‐mediated stimulation, TNF‐α (ImmunoTools GmbH, Friesoythe, Germany) at a final concentration of 100 ng ml^−1^ was added via the lid‐port‐system once daily for 6 h over three days during the resting phase. For PBMC‐mediated stimulation, isolated PBMCs were activated with 10 ng ml^−1^ 1‐Methoxy‐2‐propyl acetate (PMA) and 1 µg ml^−1^ ionomycin in RPMI for 2.5 h at 37 °C and a cell density of 10^6^ cells ml^−1^. The cells were washed in PBS and then resuspended in experimental medium and loaded onto TCC2 via the lid‐port‐system. After three days of PBMC cultivation, the cells were treated with baricitinib (Sigma–Aldrich, Munich, Germany) at a final concentration of 100 nM, which was added daily for a further three days.

### Cytotoxicity and Cell Viability Assays

Cytotoxicity Detection LDH (Sigma–Aldrich, Munich, Germany) and Cell Proliferation Reagent WST‐1 (Sigma–Aldrich, Munich, Germany) were used according to the manufacturer's instructions and as previously described.^[^ ^33^, ^47^ ^]^ To induce cell death resulting in LDH release, cells were incubated with 4% (v/v) Triton X‐100 (Sigma–Aldrich, Munich, Germany) for 24 h. Assays were performed in duplicates. ApoTox‐Glo Triplex assay (Promega Corporation, Walldorf, Germany) was performed to measure cell viability, cytotoxicity, and apoptosis using phenol red‐free DMEM (Gibco, Waltham, USA). To induce cell death, samples were incubated with 4% (v/v) Triton X‐100 for 4 h or 100 µg ml^−1^ digitonin (Boehringer Mannheim GmbH, Mannheim, Germany) for 30 min. As a positive control for apoptosis, samples were incubated with 0.1 mM camptothecin (Sigma–Aldrich, Munich, Germany) for 4 h.

### Oxygen Consumption (pO_2_) Measurement

Oxygen consumption (pO_2_) was measured using a Clark electrode (SI130 Microcathode electrode, Strathkelvin Instruments Limited, North Lanarkshire, Scotland) that was covered with a fluorinated ethylene propylene membrane (FEP, Strathkelvin Instruments Limited, North Lanarkshire, Scotland). Equilibration was performed for at least 8 h before measurement and the electrode was calibrated by a two‐point calibration with 2% (w/v) sodium sulfite solution and experimental medium saturated with atmospheric oxygen overnight. A voltage range between 400–1000 mV was used for measurements. A sample volume of 50 µl was used by adding it into the chamber and directly sealed with a polycarbonate plug. After 10 s of stable values, the obtained value was logged.

### Glucose and Lactate Measurements

To quantify glycolysis, glucose and lactate concentrations were measured daily using the Biosen C‐Line Glucose and Lactate analyzer (EKF‐diagnostic GmbH, Barleben, Germany). Calibration and validation were performed before the measurements according to the manufacturer's instructions.

### Histology, Histomorphometry, and Immunofluorescence Staining

Samples were fixed in 4% (v/v) paraformaldehyde (PFA; Electron Microscopy Sciences, Hatfield, USA) in PBS for 2 h, followed by 10%, 20%, and 30% (w/v) sucrose solution for 24 h each at 4 °C. Tissues were embedded in the SCEM embedding medium (Sectionlab, Hiroshima, Japan) and frozen by n‐hexane using acetone and dry ice as a cooling agent. Cryosections of 7 µm thickness were prepared using the Kawamoto cryofilm type 2C (Sectionlab, Hiroshima, Japan). Alcian blue staining and immunofluorescence staining were performed as previously described (antibodies listed in Table S1, Supporting Information).^[^ ^33^ ^]^ To provide quantitative data on tissue appearance, images were analyzed with FIJI ImageJ 1.52p (National Institutes of Health, Bethesda, USA). Brightness and contrast were adjusted to remove background noise. The surveyed areas were depicted in Figure S1 (Supporting Information). Gained results were normalized to the corresponding area: total area (t.a.), outer area (o.a), middle area (m.a.), and inner area (i.a.). Cell count was determined using the “Find Maxima” function, while mean fluorescence intensities were determined by threshold. The analysis pipeline was adapted to every staining.

### Enzyme‐Linked Immunosorbent Assay (ELISA)

TNF‐α and IL‐6 DuoSet ELISA were performed according to the manufacturer's instructions in duplicates (R&D Systems, Minneapolis, USA). Briefly, Nunc MaxiSorp high protein‐binding capacity 96‐well flat‐bottomed plates (Thermo Fisher Scientific, Waltham, USA) were incubated overnight at RT with the capture antibody. After washing steps, unspecific binding sites were blocked by adding reagent diluent (1% (w/v) BSA in PBS, filtrated with 0.22 µm filters (EMD Millipore Corporation, Burlington, USA, pH 7.2‐7.4) for 1 h at RT. Standards and samples were diluted and incubated for 2 h, followed by the biotinylated detection antibody and finally an incubation with Streptavidin‐HRP conjugate. Tetramethylbenzidin (TMB) Chromogen Solution was added, and the reaction was stopped after 20 min using 1 mol l^−1^ H_2_SO_4_. Absorption was measured with the Synergy HT plate reader (BioTek Instruments Inc., Winooski, USA) at 450 nm with a wavelength correction at 630 nm.

### RNA Isolation, cDNA Synthesis, and qPCR

RNA isolation, cDNA synthesis, and quantitative PCR (qPCR) were performed as previously described.^[^ ^33^ ^]^ Briefly, RNA was isolated using the RNeasy Fibrous Tissue Mini Kit (Qiagen GmbH, Hilden, Germany). The cDNA synthesis was performed using TaqMan Reverse Transcription Reagents Kit (Applied Biosystems Inc., Waltham, USA), while the Stratagene Mx3000P (Agilent Technologies Inc., Santa Clara, USA) was used for qPCR with the DyNAmo ColorFlash SYBR Green qPCR Kit (Thermo Fisher Scientific, USA). Duplicates per gene were performed with the temperature profile: 7 min initial denaturation at 95 °C, 60 cycles of 10 s at 95 °C, 7 s at 60 °C, and 9 s at 72 °C. Primer sequences were listed in Table S2 (Supporting Information).

### Statistical Analysis

GraphPad Prism Version 10.3.0 (La Jolla, San Diego, USA) was used for statistical analyses. Mann‐Whitney U test was used for direct comparisons of two independent datasets. For comparisons of more than two independent datasets, one‐way analysis using Kruskal‐Wallis with Dunn's multiple comparisons test was performed. Two‐tailed Wilcoxon matched‐pairs signed‐rank test was applied for dependent datasets. For comparisons of more than two dependent datasets, one‐way analysis using Friedman with Dunn's multiple comparisons test was performed. For two‐way analysis, the mixed‐effect analysis with Šídák's multiple comparisons test was performed. The statistical tests used are indicated in the figure legends. Pvalues of <0.05 were considered statistically significant (*p <0.05, **p <0.01, ***p <0.005). Data are shown as box plots (centerline, median; box limits, upper and lower quartiles; whiskers, maximum and minimum) if not indicated otherwise.

## Conflict of Interest

M. Herrmann and T. Leeuw are employees of Sanofi‐Aventis Deutschland GmbH, which partly supported the study. The other authors declare that the research was conducted in the absence of any commercial or financial relationships that could be construed as a potential conflict of interest.

## Author Contributions

F.B. and T. G. contributed equally to this work. A.D., T.G. and F.B. performed conceptualization; A.D., D.H.D.N, C.L., and K.R. performed methodology; A.D., D.H.D.N., C.L., and K.R. performed formal analysis; A.D., T.G., K.R., and M.P. performed validation; A.D., and T.G. performed investigation; A.D., and K.R. performed data curation; A.D., K.R, M.P., T.G., and F.B. wrote the original draft preparation; A.D., D.H.D.N., C.L., K.R., M.P., G.K., T.L., M.H., T.G., and F.B. wrote—reviewed and edited; A.D. performed visualization; A.D., T.L., T.G., F.B. performed supervision; A.D., T.L., M.H., T.G., and F.B. performed project administration; A.D., T.G., and F.B. performed funding acquisition. All authors have read and agreed to the published version of the manuscript.

## Supporting information



Supporting Information

## Data Availability

The data that support the findings of this study are available on request from the corresponding author. The data are not publicly available due to privacy or ethical restrictions.
