# Supplementary material for: Microphysiological System‐Generated Physiological Shear Forces Reduce TNF‐α‐Mediated Cartilage Damage in a 3D Model of Arthritis
Source: Adv Sci (Weinh). 2024 Dec 24;12(7):2412010. doi: 10.1002/advs.202412010 (PMC11831510; doi:10.1002/advs.202412010)
Supplement: Supplementary file 1 — Supporting Information [file ADVS-12-2412010-s001.docx]

**Supplementary Tables**

Table S1: List of primary and secondary antibodies and kits used for immunofluorescence.


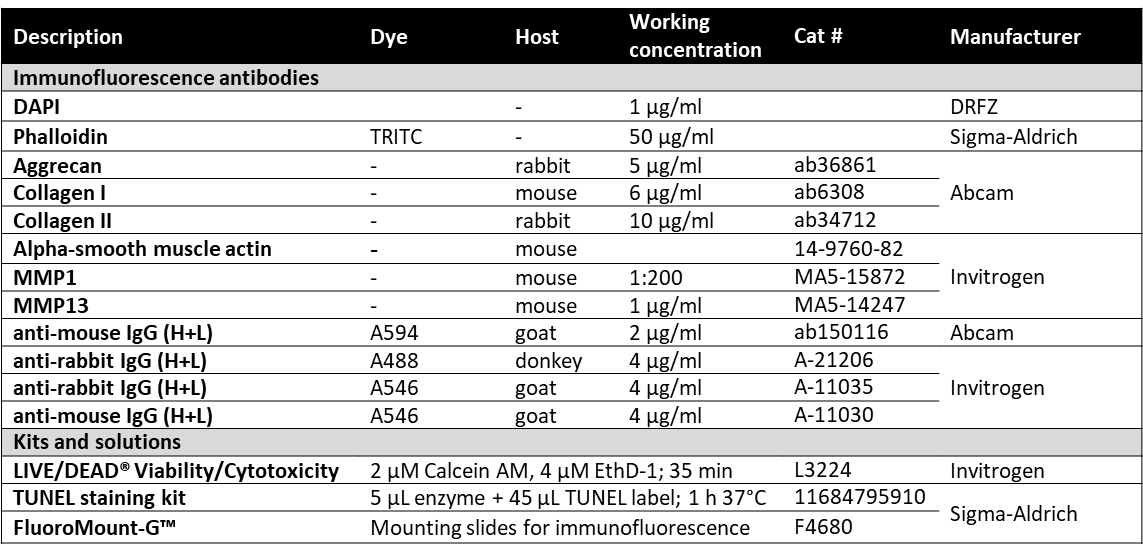


Table S2: Primer sequences.


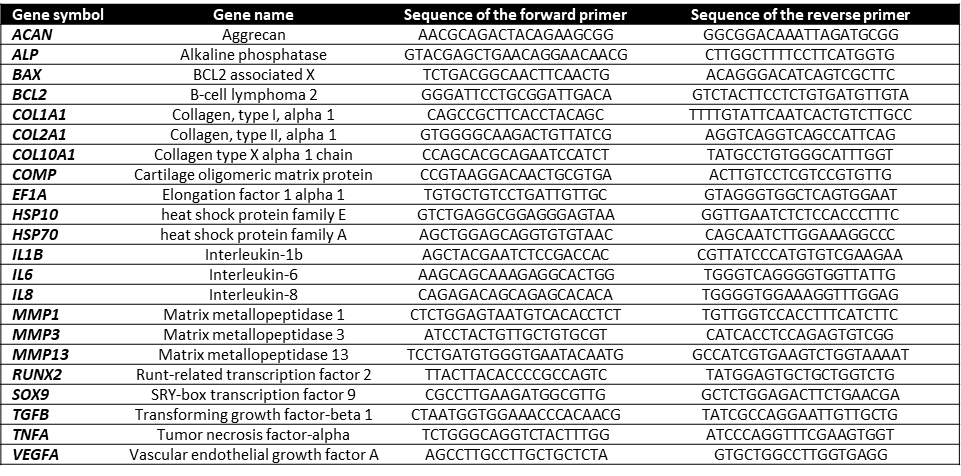


Table S3: Values to calculate the flow pattern and fluidic shear forces within the flow chamber.


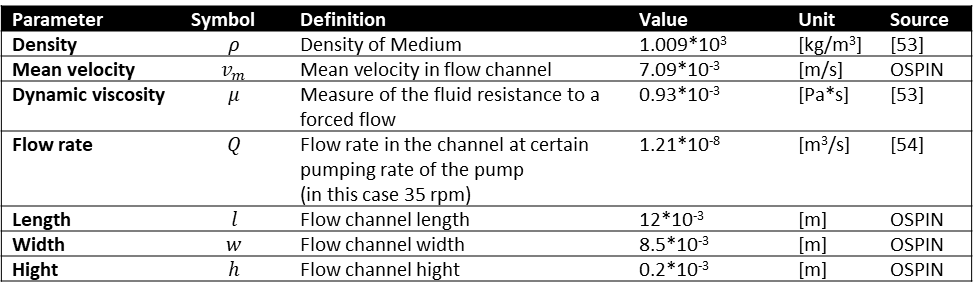


**Supplementary Figures**


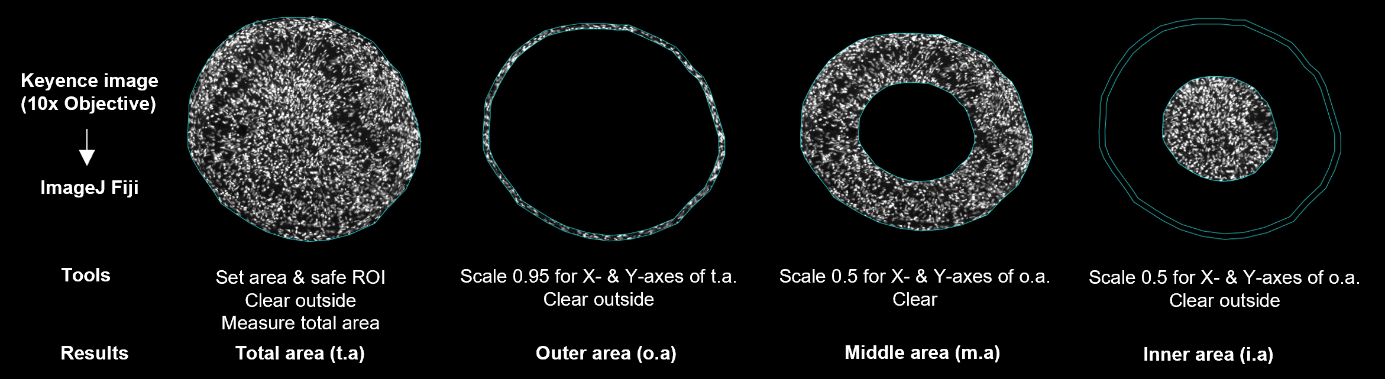


Figure S1: Image analysis pipeline using FIJI ImageJ.


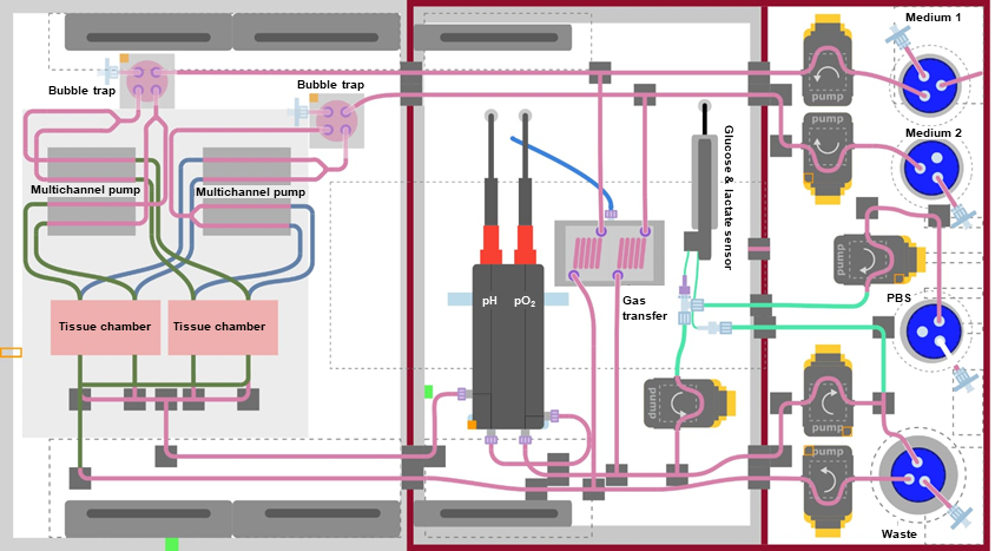


Figure S2: Overview of our bioreactor platform. To cover as many applications as possible, the bioreactor has several functionalities. Unused parts are greyed. Medium from the medium reservoir in the bubble trap is pumped through the multichannel pump into the chamber. The tissue chamber is placed on a heating plate to keep the temperature stable. Leaving the tissue chamber the medium passes the pH and pO_2_ probes for process monitoring and flows through the gas transfer chamber with a gas-permeable tubing. A controlled air and CO_2_ flow regulates the process pH. Finally, the medium drops back into the bubble trap preventing bubble accumulation in the circular flow.


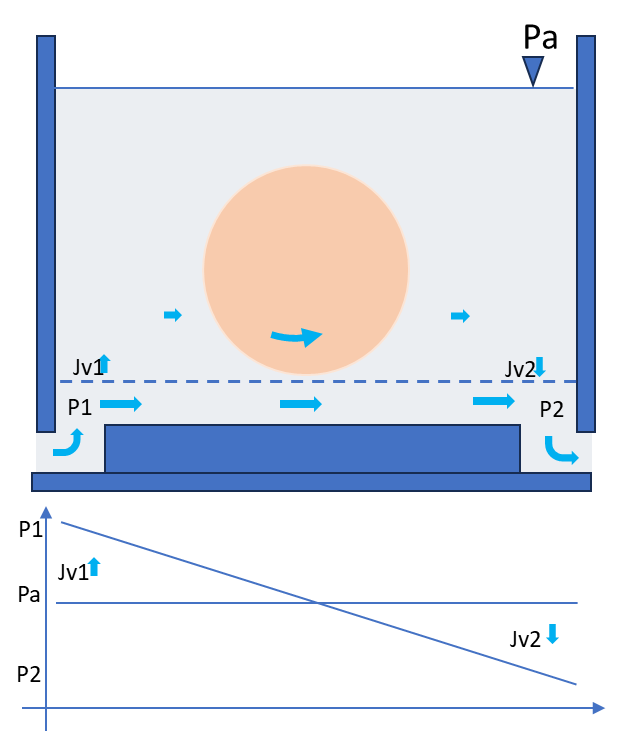


Figure S3: Schematic overview of medium flow and pressure differences at the flow area and within the TCC1.


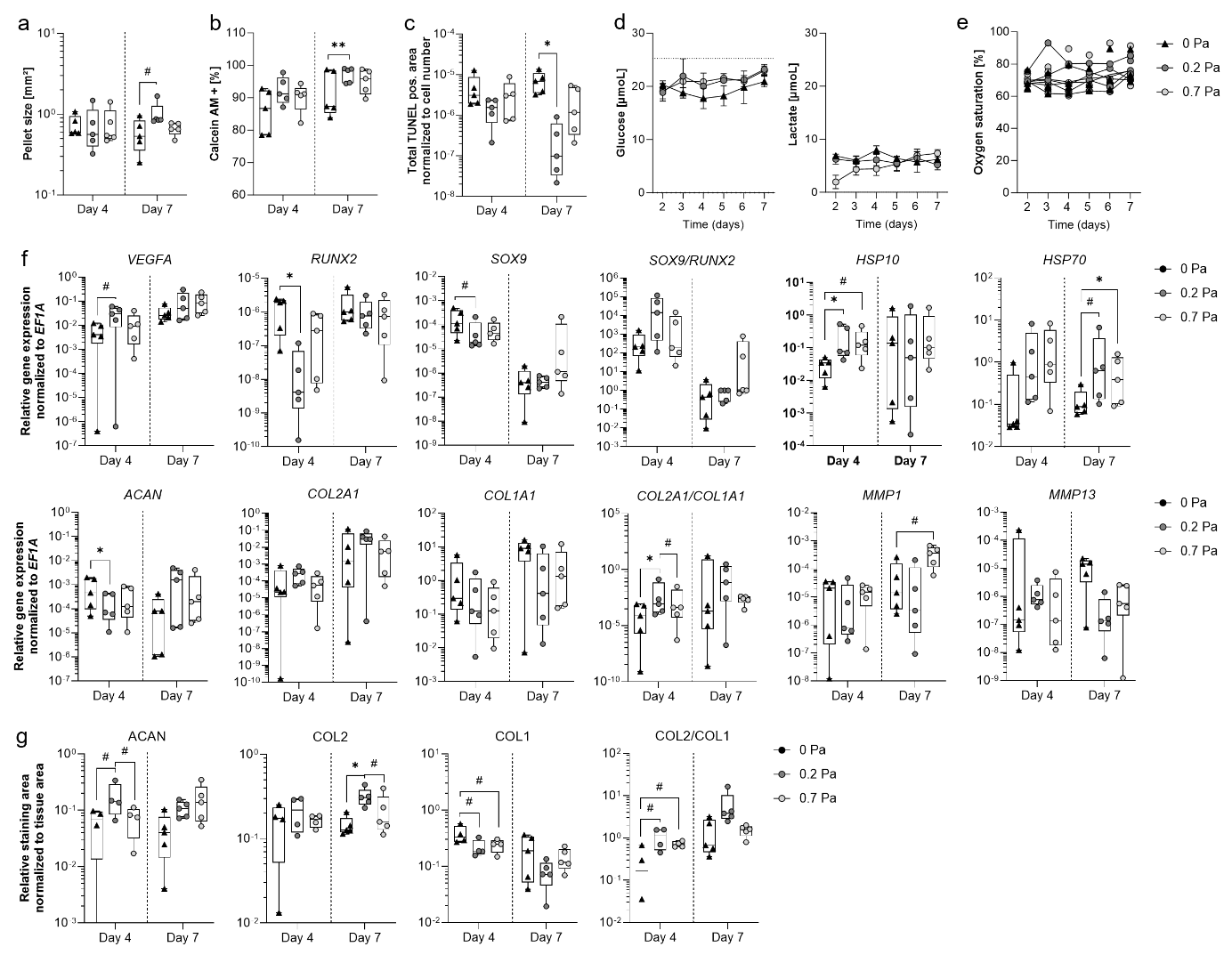


*Figure S4: Effect of various shear stresses on CMC viability, gene and protein expression. Comparison of the cultivation procedures after 4 and 7 days: 0 rpm (0 Pa) versus dynamic cultivation at physiological and pathophysiological high shear stress using 35 rpm (0.2 Pa) and 100 rpm (0.7 Pa), respectively. (a) Measurement of the CMC size in the culture compartment. The area in mm² was determined using ImageJ and represents the mean of two randomly selected CMCs per condition (n=5). (b) Percentage of CMC stained with Calcein-AM. Staining was quantified using ImageJ (n=5). (c) Apoptotic cells present in the CMC normalized to the cell number. TUNEL staining was performed normalized to DAPI staining using ImageJ (n=5). (d) Glucose and lactate concentration in the MPS supernatant over the course of the experiment. Values were measured using the Biosen C-line analyzer (n=5). The control (dotted line: mean glucose = 25.25 mmol/l; mean lactate = 0.24 mmol/l) shows glucose and lactate concentration within the cell-free culture medium. (e) Oxygen saturation of the culture medium over time. Daily measurement of oxygen saturation using a Clark electrode with n=5. (f) Relative expression of anabolic and catabolic marker genes normalized to the housekeeper EF1A (n=5). (g) Comparing chondrogenic differentiation on protein level. Quantification of the alcian blue staining was performed using ImageJ. Data are shown as box plots (center line, median; box limits, upper and lower quartiles; whiskers, maximum and minimum values; all data points).* *Statistical analysis was performed using a mixed-effects model with the Geisser-Greenhouse correction and Tukey's multiple comparisons test. P-values are indicated in the graphs with #p < 0.1, *p < 0.05, **p < 0.01, ****p < 0.0001.*

Compared to pathophysiological low stress of 0 Pa, a physiological fluidic shear stress of 0.2 Pa enhanced CMC size by trend and significantly improved viability, while reducing apoptosis (Figure S4a-c). These positive effects were lost again under pathophysiological high fluidic shear stress of 0.7 Pa. Glucose uptake, lactate secretion, and oxygen saturation were not affected by the shear stresses applied within 7 days (Figure S4d, e). Notably, oxygen saturation was continuously monitored during the entire culture period (Figure S4e) and was not affected by different flow regiments. Next, we analyzed the hMSC-derived pre-CMCs by looking at gene expression of anabolic cartilage markers (*SOX9, SOX9*/*RUNX2, ACAN, COL2, COL2/COL1*) and catabolic cartilage markers (*MMP1, MMP13*) by RT-qPCR (Figure S4f). The effect of shear forces at 0.2 Pa trended to increase the expression of *VEGFA* and decrease the expression of *RUNX2* and *SOX9*, while the ratio between *SOX9* and *RUNX2* increased at day 4 compared to 0 Pa and 0.7 Pa. In addition, cultivation at 0.2 Pa increased the ratio between chondroanabolic *COL2A1* and *COL1A1* expression at day 4 compared to 0 Pa and 0.7 Pa but did not lead to an increase in the catabolic gene *MMP1* at day 7, as also observed in the 0.7 Pa condition. On day 7 shear forces of 0.2 Pa and 0.7 Pa numerically decreased *MMP13* expression as compared to 0 Pa. However, the expression levels on day 7 were almost identical under all conditions (Figure S4f). *HSP10* and *HSP70* are rapidly induced and overexpressed, which is a well-known phenomenon in response to stress stimuli. On the protein level, shear forces of 0.2 Pa lead to higher expression of aggrecan and an increased ratio of type 2/type 1 collagen compared to 0 Pa and 0.7 Pa (Figure S4g).


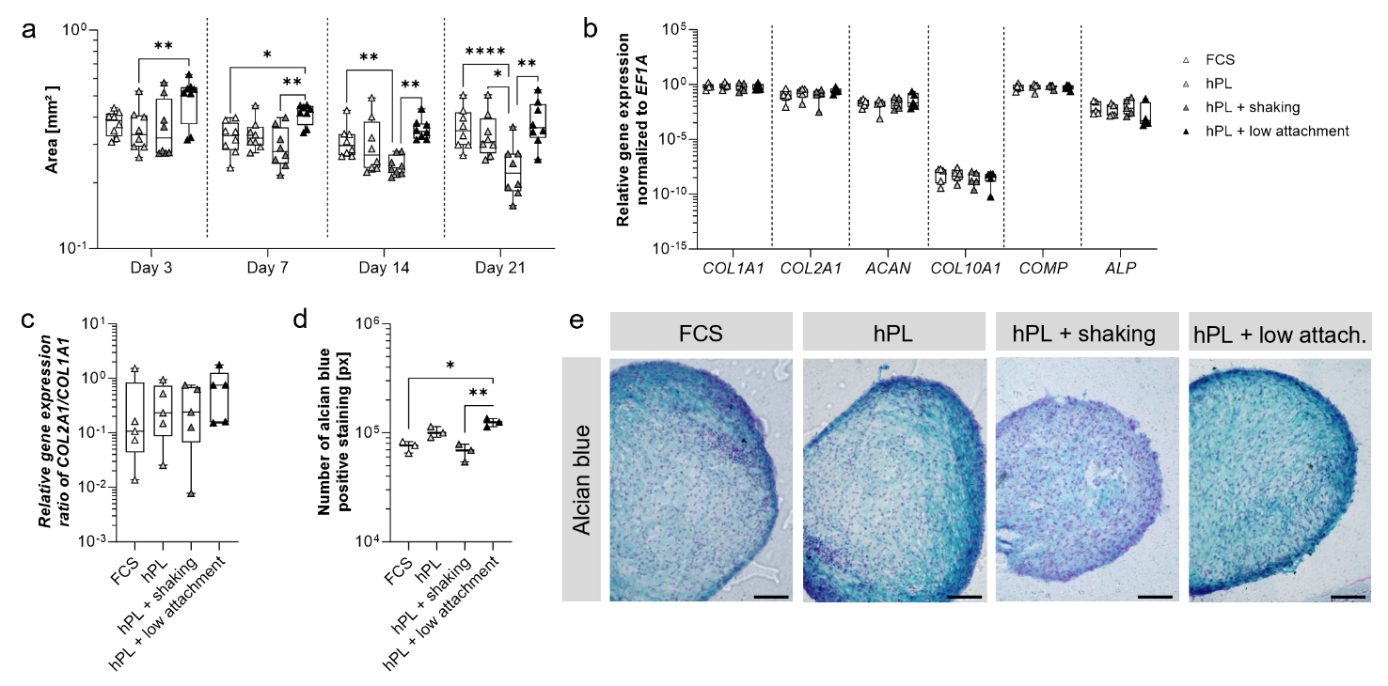


*Figure S5: Culture conditions influence differentiation of in vitro 3D CMC formation. (a) Measurement of the size of CMC constructs comparing various culture conditions. The area in mm² was determined using ImageJ and represents the mean of two randomly selected CMCs per condition. (b) Relative expression of chondrogenic marker genes and (c) the ratio of COL2A1 to COL1A1 normalized to the housekeeper EF1A were not significantly altered by the culture conditions after 21 days of differentiation. (d) Comparing chondrogenic differentiation on protein level, quantification of the alcian blue staining was performed using ImageJ. (e) Representative images of the alcian blue and nuclear fast red staining after 21 days of differentiation. Scale bar shows 100 μm. Data are shown as box plots (center line, median; box limits, upper and lower quartiles; whiskers, maximum and minimum values; all data points). Statistics: Two-way ANOVA and RM one-way ANOVA Tukey's multiple comparisons test; p-values are indicated in the graphs with *p < 0.05, **p < 0.01, ****p < 0.0001.*

To further improve the formation and differentiation of CMCs and to provide a fully animal-free cell culture setup, we tested different parameters, namely shaking, the use of hPL instead of fetal calf serum (FCS), and the incubation in ULA-plates. CMCs became significantly denser over time when the cultures were shaken at a frequency of 0.5 Hz (Figure S5a). To evaluate the effects of serum and ULA plates on the chondrogenic differentiation processes, we analyzed the gene expression of *COL1A1*, *COL2A1*, *ACAN*, *COMP*, and *COL10A1* while *ALP* served as an indicator for cellular hypertrophy (Figure S5b). hPL performed better than FCS and was subsequently used. When using hPL in ULA-plates during the initial CMC formation step, we observed a numerically reduced *ALP* expression (Figure S5b), a higher ratio of *COL2A1* to *COL1A1* (Figure S5c), and a distinct increase in glycosaminoglycan deposition indicating optimized chondrogenic culture conditions (Figure S5d, e).


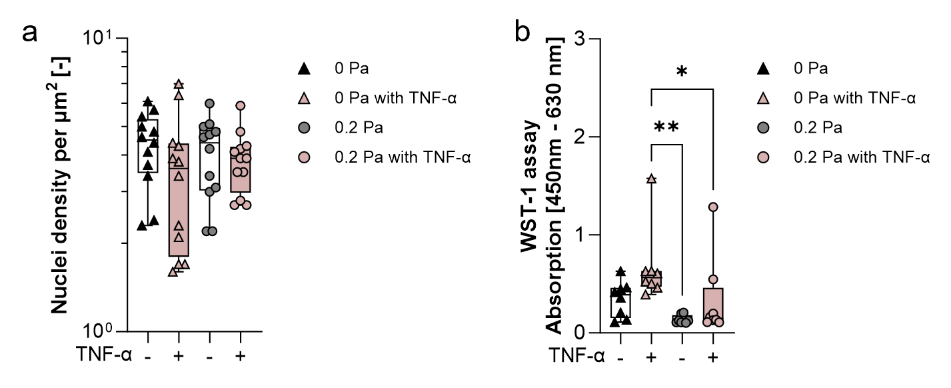


Figure S6: Short-term exposure of 100 ng/ml TNF-α for three days reduced nuclei density and perfused cultivation lowers metabolic activity. (a) Nuclei density per μm^2^ for 5 (n=12). (b) WST-1 assay was performed on day 21 without and with three days of TNF-α stimulation either with 0 rpm (0 Pa) or 35 rpm (0.2 Pa) pump rates (n=8). The NADPH-dependent formazan production was measured at 450 nm (reference wavelength: 630 nm). Neg. Ctrl: treatment with 4% Triton X-100 for 24 h.


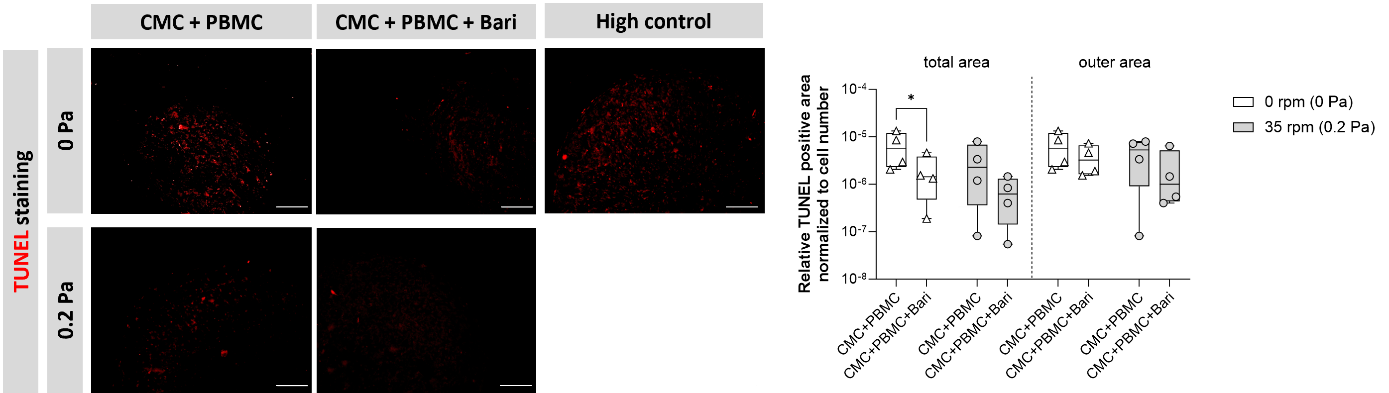


Figure S7: TUNEL staining was performed on 7 µm CMC sections (n=4) and quantified by normalizing TUNEL to the cell number (DAPI staining) using ImageJ.
